# Semi-Infinite Structure Analysis with Bimodular Materials with Infinite Element

**DOI:** 10.3390/ma15020641

**Published:** 2022-01-15

**Authors:** Wang Huang, Jianjun Yang, Jan Sladek, Vladimir Sladek, Pihua Wen

**Affiliations:** 1 School of Traffic and Transportation Engineering, Changsha University of Science and Technology, Changsha 410114, China; huangwang1003@gmail.com (W.H.); yang@csust.edu.cn (J.Y.); 2 Institute of Construction and Architecture, Slovak Academy of Sciences, 84503 Bratislava, Slovakia; jan.sladek@savba.sk (J.S.); vladimir.sladek@savba.sk (V.S.); 3 School of Engineering and Materials Science, Queen Mary University of London, London E1 4NS, UK

**Keywords:** meshless method, finite block method, semi-infinite structure, bimodular material, mapping technique, infinite element

## Abstract

The modulus of elasticity of some materials changes under tensile and compressive states is simulated by constructing a typical material nonlinearity in a numerical analysis in this paper. The meshless Finite Block Method (FBM) has been developed to deal with 3D semi-infinite structures in the bimodular materials in this paper. The Lagrange polynomial interpolation is utilized to construct the meshless shape function with the mapping technique to transform the irregular finite domain or semi-infinite physical solids into a normalized domain. A shear modulus strategy is developed to present the nonlinear characteristics of bimodular material. In order to verify the efficiency and accuracy of FBM, the numerical results are compared with both analytical and numerical solutions provided by Finite Element Method (FEM) in four examples.

## 1. Introduction

It has been shown that certain materials such as composites, porous materials, rocks, cement concrete and asphalt concrete, etc., show significant differences in their strength in tension and compression states. The modulus of elasticity as well as the Poisson’s ratio of the material may also change under tensile and compressive states [1,2,3]. Take the concrete material as an example; the compressive modulus is about 1.5~2 times the tensile modulus [4,5,6]. So, for an accurate numerical simulation, this characteristic of material has to be considered. It constructs a typical material nonlinear model.

In order to evaluate bearing capacity and stability, the civil structure with the soil–foundation interaction is commonly investigated numerically, including airport runways, highway pavement, stacking dock, mineral deposit, geotechnical slope and so on. The soil medium is simplified as an infinite or semi-infinite domain. The most common approach with FEM is to use massive elements to simulate an unbounded domain. The application of large-scale finite element discretization could result in an increase in computational burden [7]. Furthermore, the inaccurate results could be obtained due to the truncated boundaries in the numerical procedure. To overcome this difficulty, the Boundary Integral Equations Method (BIEM), also known as the Boundary Element Method (BEM), is coupled with the FEM [8,9]. However, it is difficult to derive the fundamental solutions in general cases, especially for non-homogeneous and nonlinearity of materials. Meanwhile, the semi-analytical finite element method was developed to reduce the time cost of 3D model simulation [10,11] and applied in pavement structural analysis [12,13], but it mainly focuses on linear analysis or problems without complicated loads. The unbounded problems can be overcome by introducing mapped infinite elements, i.e., utilizing the infinite element to extend the FEM to unbounded domain problems [14,15,16,17]. The shape function describes the far-field characteristic of the problem, which can be obtained using mapping to transform the global infinite region into a local finite domain by Bettess et al. [17,18,19,20]. As an alternative, these issues can be solved with the meshless methods coupling with an infinite-mapping technique [7].

**Figure 1:**
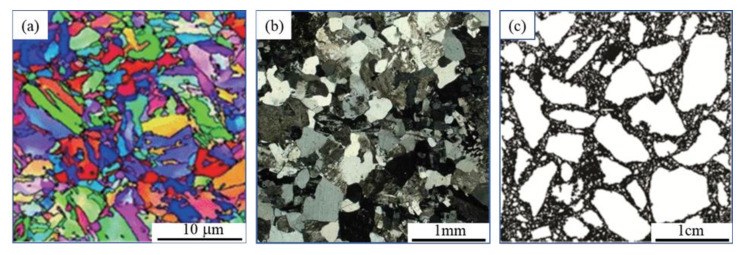
Scanning images of solid materials at different scales: (**a**) twin structure of carbon steel; (**b**) fine grain structure of granite; (**c**) meso structure of concrete.

In engineering analysis, the linear elasticity of material is not valid for general issue. The material mechanical properties are closely related to their micro structure. The scanning images of the building materials are shown in Figure 1 and present similar mottled patterns at different scales. The heterogeneity is manifested in the micro-scale for the metal materials, and its mechanical properties accord with the linear elastic hypothesis. For the rock or concrete materials, their heterogeneity is displayed in the mesoscale and the assumption of linear elasticity sometimes produces computational errors which cannot be ignored.

Commercial numerical software in engineering, including ABAQUS, are widely used in engineering and manufacturing. However, it is still a challenging task to solve bimodular problems efficiently [21,22,23,24,25,26]. Nevertheless, the development of new numerical methods is always attractive to solve difficult and complicated engineering problems. Unlike the traditional numerical method, the computational framework of the meshless method was based on the scattered nodes. In the 1990s, the meshless method was developed based upon the Galerkin method. In 1992, the diffuse element method (DEM) was proposed by Nayroles et al. [27]. The Moving-Least Square (MLS) method was introduced to construct the meshless shape functions with Galerkin method in numerical discretization. In 1994, Belytschkoet al. presented the Element-Free Galerkin method (EFGM) [28], in which Lagrange was employed to ensure the boundary conditions were being satisfied. Since then, the EFGM has been widely used to simulate the fracture failure of materials and to show its superiority over the traditional FEM [29,30]. In 1996, Belytschko et al. published a comprehensive review [31] which attracted exclusive attention in computational mechanics. This can be regarded as the beginning of the meshless method in numerical engineering. Another important development was the introduction of the local weak form methods. In 1998, Atluri et al. proposed the Meshless Local Petrov-Galerkin (MLPG) method [32]. The discrete system equation is based on a nodal assembly with more conciseness in numerical implementation. In 1995, Liu et al. proposed a Reproducing Kernel Particle Method (RKPM) approximation [33,34,35]. Thereafter, several meshless methods were developed such as the Method of Fundamental Solution (MFS) [36,37,38], the local Radial Point Interpolation Method (RPIM) [39,40,41], the local Radial Basis Function (RBF) collocation method [42,43,44] and the Meshless Intervention-Point (MIP) method [45], etc. In 2014, Wen et al. proposed the meshless FBM [46]. In the finite block method, the mapping technique is implemented numerically with the infinite elements for the infinite domain problems [7]. Afterwards, the FBM is successfully applied to nonlinear elasticity problems, contact problems and heat conduction problems [47,48,49]. It has been demonstrated in the analysis of bimodular problems for two-dimensional problems [50].

In this paper, the FBM is extended to three-dimensional semi-infinite structures in bimodular materials. The infinite block mapping technique is introduced to present the semi-infinite structure and implemented with the meshless finite block method to construct the intrinsic constitutive equations in iterative analysis. The meshless finite block method with the infinite block mapping technique is formulated for 3D bimodular problems. The FEM solution is considered as a benchmark for numerical analysis, and the accuracy of the proposed method is observed by ABAQUS with subroutine UMAT developed for bimodular materials.

## 2. Bimodular Material Constitutive Equations

**Figure 2:**
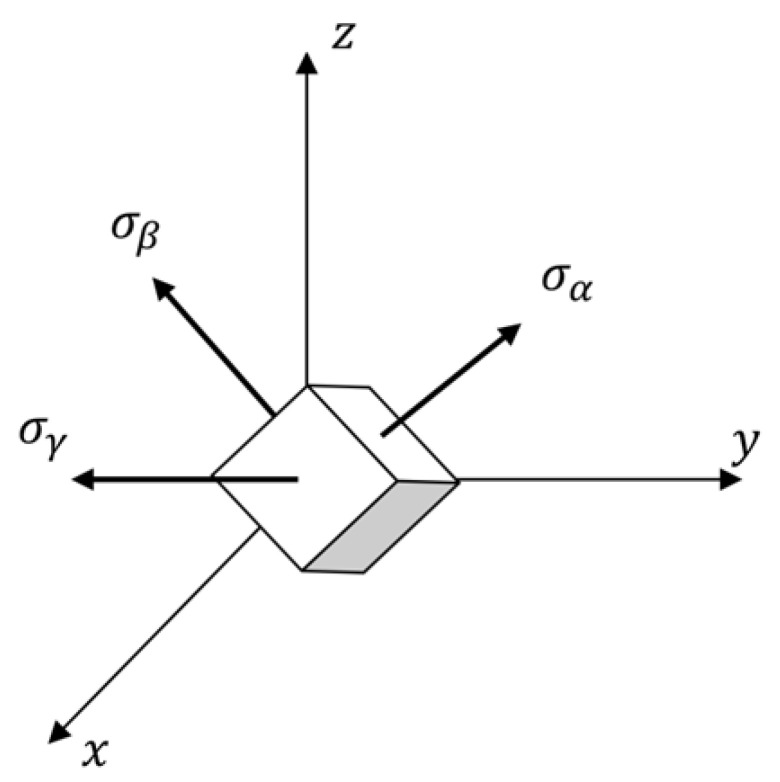
Principal stresses and their direction in Cartesian’s coordinate system.

Suppose $\sigma_{\alpha },\sigma_{\beta }$ and $\sigma_{\gamma }$ are principal stresses, as shown in Figure 2. The generalized Hooke’s law, in matrix form, as

(1) $$\tilde{\varepsilon }=A\tilde{\sigma }\;\cdots \mathrm{or}\cdots \;\tilde{\sigma }=Q_{I}\tilde{\varepsilon }$$

where **A** is the flexibility matrix, **Q**_I_ is the elasticity matrix, $\tilde{\varepsilon }$ is the nodal strain vector in the principal directions and $\tilde{\sigma }$ is the nodal stress vector in the principal directions, which are defined as

(2) $$A=\left(\begin{array}{cccccc} a_{11} & a_{12} & a_{13} & 0 & 0 & 0\\ a_{21} & a_{22} & a_{23} & 0 & 0 & 0\\ a_{31} & a_{32} & a_{33} & 0 & 0 & 0\\ 0 & 0 & 0 & a_{44} & 0 & 0\\ 0 & 0 & 0 & 0 & a_{55} & 0\\ 0 & 0 & 0 & 0 & 0 & a_{66} \end{array}\right),Q_{I}=A^{-1}$$

(3) $$\tilde{\varepsilon }={\left\{\begin{array}{cccccc} \varepsilon_{\alpha }, & \varepsilon_{\beta }, & \varepsilon_{\gamma }, & \varepsilon_{\beta \gamma }, & \varepsilon_{\alpha \gamma }, & \varepsilon_{\alpha \beta } \end{array}\right\}}^{T}$$

(4) $$\tilde{\sigma }={\left\{\begin{array}{cccccc} \sigma_{\alpha }, & \sigma_{\beta }, & \sigma_{\gamma }, & \sigma_{\beta \gamma }, & \sigma_{\alpha \gamma }, & \sigma_{\alpha \beta } \end{array}\right\}}^{T}$$

With the analytical theory proposed by Ambartsumyan and complemented with shear moduli [1,21,22], it is assumed that $a_{ij}=-v^{-}/E^{-}=-v^{+}/E^{+}$, $a_{jj}=1/E^{+}$ or $1/E^{-}$, (*I* = 1, *j* = 1, 2, 3), where $E^{+}$ and $E^{-}$ present as the tensile and compressive moduli respectively, $v^{+}$ and $v^{-}$ are the tensile and compressive Poisson’s ratio, respectively; $a_{44}=1/G_{\beta \gamma }$, $a_{55}=1/G_{\alpha \gamma }$, $a_{66}=1/G_{\alpha \beta }$, in which, $G_{\beta \gamma }$, $G_{\alpha \gamma }$ and $G_{\alpha \beta }$ are the shear moduli. The shear stresses or strains in the principal directions are zero. According to the shear moduli algorithm [13], it is assumed that the axes *x*, *y* and *z* tend to axes α, β and γ, respectively. Then, we have

(5) $$\begin{array}{l} G_{\beta \gamma }=\underset{\begin{array}{l} l_{1},m_{2},n_{3}\to 1\\ l_{2},l_{3},m_{1},m_{3},n_{1}n_{2}\to 0 \end{array}}{lim}G_{yz}=\underset{\begin{array}{l} l_{1},m_{2},n_{3}\to 1\\ l_{2},l_{3},m_{1},m_{3},n_{1}n_{2}\to 0 \end{array}}{lim}\frac{\tau_{yz}}{\gamma_{yz}}\\ G_{\alpha \gamma }=\underset{\begin{array}{l} l_{1},m_{2},n_{3}\to 1\\ l_{2},l_{3},m_{1},m_{3},n_{1}n_{2}\to 0 \end{array}}{lim}G_{xz}=\underset{\begin{array}{l} l_{1},m_{2},n_{3}\to 1\\ l_{2},l_{3},m_{1},m_{3},n_{1}n_{2}\to 0 \end{array}}{lim}\frac{\tau_{xz}}{\gamma_{xz}}\\ G_{\alpha \beta }=\underset{\begin{array}{l} l_{1},m_{2},n_{3}\to 1\\ l_{2},l_{3},m_{1},m_{3},n_{1}n_{2}\to 0 \end{array}}{lim}G_{xy}=\underset{\begin{array}{l} l_{1},m_{2},n_{3}\to 1\\ l_{2},l_{3},m_{1},m_{3},n_{1}n_{2}\to 0 \end{array}}{lim}\frac{\tau_{xy}}{\gamma_{xy}} \end{array}$$

There are three cases to obtain $G_{\beta \gamma }$, $G_{\alpha \gamma }$ and $G_{\alpha \beta }$,

(1) If all three principal stresses are equal, i.e., $\sigma_{\alpha }=\sigma_{\beta }=\sigma_{\gamma }$, we have
  a. If $\sigma_{\alpha }\leq 0$, then
    (6) $$G_{\beta \gamma }=G_{\alpha \gamma }=G_{\alpha \beta }=G^{-}=\frac{E^{-}}{2(1+v^{-})}$$
  b. If $\sigma_{\alpha }>0$, then
    (7) $$G_{\beta \gamma }=G_{\alpha \gamma }=G_{\alpha \beta }=G^{+}=\frac{E^{+}}{2(1+v^{+})}$$

(2) If only two of the three principal stresses are equal, i.e., $\sigma_{\alpha }=\sigma_{\beta }\neq \sigma_{\gamma }$, we hold
  (8) $$G_{\alpha \beta }=\frac{\sigma_{\alpha }-\sigma_{\gamma }}{2(\varepsilon_{\alpha }-\varepsilon_{\gamma })}=G_{\alpha \gamma }=G_{\beta \gamma }$$

(3) If all three principal stresses are not equal, i.e., $\sigma_{\alpha }\neq \sigma_{\beta }\neq \sigma_{\gamma }$, we have
  (9) $$G_{\beta \gamma }=\frac{\sigma_{\beta }-\sigma_{\gamma }}{2(\varepsilon_{\beta }-\varepsilon_{\gamma })},G_{\alpha \gamma }=\frac{\sigma_{\alpha }-\sigma_{\gamma }}{2(\varepsilon_{\alpha }-\varepsilon_{\gamma })}\;\mathrm{and}\;G_{\alpha \beta }=\frac{\sigma_{\alpha }-\sigma_{\beta }}{2(\varepsilon_{\alpha }-\varepsilon_{\beta })}$$

In the Cartesian coordinate system, the directional cosines for each principal strain are defined as

(10) $$\begin{array}{l} \alpha =\left(l_{1},l_{2},l_{3}\right)\\ \beta =\left(m_{1},m_{2},m_{3}\right)\\ \gamma =\left(n_{1},n_{2},n_{3}\right) \end{array}$$

The strain vector in different coordinate systems is obtained, in matrix form, as

(11) $$\tilde{\varepsilon }=L\varepsilon ,$$

where ε is the strain vector in Cartesian’s coordinate system and **L** is the transformation matrix defined by

(12) $$L=\left(\begin{array}{cccccc} l_{1}{}^{2} & m_{1}{}^{2} & n_{1}{}^{2} & m_{1}n_{1} & l_{1}n_{1} & l_{1}m_{1}\\ l_{2}{}^{2} & m_{2}{}^{2} & n_{2}{}^{2} & m_{2}n_{2} & l_{2}n_{2} & l_{2}m_{2}\\ l_{3}{}^{2} & m_{3}{}^{2} & n_{3}{}^{2} & m_{3}n_{3} & l_{3}n_{3} & l_{3}m_{3}\\ 2l_{2}l_{3} & 2m_{2}m_{3} & 2n_{2}n_{3} & m_{2}n_{3}+m_{3}n_{2} & l_{2}n_{3}+l_{3}n_{2} & l_{2}m_{3}+l_{3}m_{2}\\ 2l_{1}l_{3} & 2m_{1}m_{3} & 2n_{1}n_{3} & m_{1}n_{3}+m_{3}n_{1} & l_{1}n_{3}+l_{3}n_{1} & l_{1}m_{3}+l_{3}m_{1}\\ 2l_{1}l_{2} & 2m_{1}m_{2} & 2n_{1}n_{2} & m_{1}n_{2}+m_{2}n_{1} & l_{1}n_{2}+l_{2}n_{1} & l_{1}m_{2}+l_{2}m_{1} \end{array}\right)$$

The strain energy density *U* in terms of the principal strains and elastic matrix, at each node, yields

(13) $$U=\frac{1}{2}{\tilde{\varepsilon }}^{T}Q_{I}\tilde{\varepsilon }=\frac{1}{2}\varepsilon^{T}L^{T}Q_{I}L\varepsilon$$

Therefore, the elastic matrix **Q** in Cartesian’s coordinate system is obtained by

(14) $$Q=L^{T}Q_{I}L$$

## 3. The Meshless Finite Block Method

### 3.1. Lagrange Polynomial Interpolation

**Figure 3:**
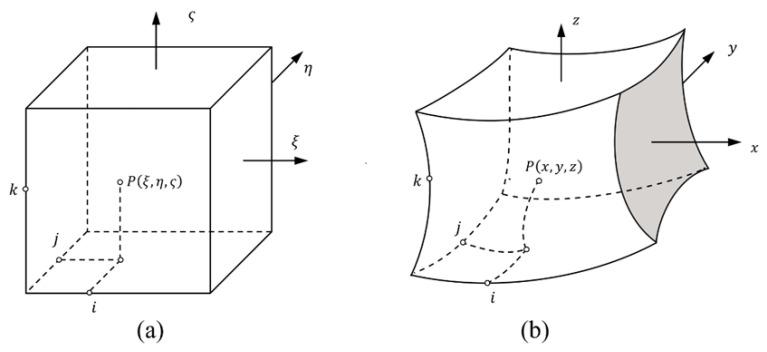
Mapping technique for finite block method: (**a**) normalized domain; (**b**) physical domain.

Consider a 3D square in normalized domain mapping to the physical domain, as shown in Figure 3. The Lagrange polynomials in the coordinate system (ξ,η,ζ) are used to interpolate function *u*

(15) $$u(\xi ,\eta ,\zeta )=\sum_{i=1}^{N_{\xi }}\sum_{j=1}^{N_{\eta }}\sum_{k=1}^{N_{\zeta }}F(\xi ,\xi_{i})G(\eta ,\eta_{j})H(\zeta ,\zeta_{k})u_{p}$$

where $u_{p}$ indicates the nodal value, subscript *p* denotes the number of node at *P*$\left(\xi_{i},\eta_{i},\zeta_{i}\right)$ in the global system and functions

(16) $$F(\xi ,\xi_{i})=\prod_{\begin{array}{l} m=1\\ m\neq i \end{array}}^{N_{\xi }}\frac{\xi -\xi_{m}}{\xi_{i}-\xi_{m}},G(\eta ,\eta_{j})=\prod_{\begin{array}{l} m=1\\ m\neq j \end{array}}^{N_{\xi }}\frac{\eta -\eta_{m}}{\eta_{j}-\eta_{m}},F(\zeta ,\zeta_{k})=\prod_{\begin{array}{l} m=1\\ m\neq k \end{array}}^{N_{\xi }}\frac{\zeta -\zeta_{m}}{\zeta_{k}-\zeta_{m}}$$

where $N_{\xi }$, $N_{\eta }$ and $N_{\zeta }$ denote the numbers of node distributed along the axes ξ, η and ζ, respectively. The shape function is obtained simply as

(17) $$\varphi_{p}(\xi ,\eta ,\zeta )=F(\xi ,\xi_{i})G(\eta ,\eta_{j})H(\zeta ,\zeta_{k})$$

The partial differential with respect to axis ξ can be obtained directly

(18) $$\frac{\partial \varphi_{p}}{\partial \xi }=\frac{\partial F(\xi ,\xi_{i})}{\partial \xi }G(\eta ,\eta_{j})H(\zeta ,\zeta_{k})=\frac{\sum_{m=1}^{N_{\xi }}\prod_{l=1,l\neq m}^{N_{\xi }}\left(\xi -\xi_{l}\right)}{\prod_{m=1,m\neq i}^{N_{\xi }}\left(\xi_{i}-\xi_{m}\right)}G(\eta ,\eta_{j})H(\zeta ,\zeta_{k})$$

### 3.2. Partial Differential Matrix

The partial derivative of function *u* in Equation (15) can be arranged in a vector. For example, the nodal first order partial derivative of function *u* can be written, in the vector form, as

(19) $$\begin{array}{cc} \begin{array}{l} u_{,\alpha }=U_{\alpha }=D_{\alpha }u,D_{\alpha }={\left\{\varphi_{ijk,\alpha }\right\}}_{M\times M}\\ p=p(i,j,k),(i=1,2,\ldots ,N_{\xi },j=1,2,\ldots ,N_{\eta },k=1,2,\cdots ,N_{\varsigma };\alpha =\xi ,\eta ,\zeta ) \end{array} & \end{array}$$

where *p* is the number of node *P*(*i, j, k*) in the global system; $M(=N_{\xi }\times N_{\eta }\times N_{\zeta })$ indicates the number of nodes in the local coordinate system,

(20) $$\begin{array}{cc} u_{,\alpha }=\left\{\frac{\partial u}{\partial \alpha }\right\},u={\left\{u_{1},u_{2},\cdots ,u_{M}\right\}}^{T},u_{p}={\left\{u_{x}^{\left(p\right)},u_{y}^{\left(p\right)},u_{z}^{\left(p\right)}\right\}}^{T}, & \alpha =\xi ,\eta ,\varsigma \end{array}$$

and

(21) $$D_{\alpha }=\left\{\frac{\partial \varphi_{1}}{\partial \alpha },\frac{\partial \varphi_{2}}{\partial \alpha },\cdots ,\frac{\partial \varphi_{M}}{\partial \alpha }\right\}$$

In addition, the *L*-th order partial derivative with respect to the coordinates ξ, η and ζ can be approximated as

(22) $$u_{,\xi \eta \zeta }^{\left(lmn\right)}=\frac{\partial^{l+m+n}u}{\partial \xi^{l}\partial \eta^{m}\partial \zeta^{n}},l+m+n=L$$

Therefore, the higher-order partial differentials in Equation (22) can be obtained, in terms of the first-order partial derivative matrices $D_{\xi }$, $D_{\eta }$ and $D_{\zeta }$, as

(23) $$u_{,\xi \eta \zeta }^{\left(lmn\right)}\approx D_{\xi }^{l}D_{\eta }^{m}D_{\zeta }^{n}u$$

### 3.3. Mapping Differential Matrix

For three-dimensional problems, a hexahedron block with 20 seeds is selected in order to transform the coordination (*x*, *y*, *z*) to (ξ,η,ζ) as shown in Figure 3. The mapping function is expressed as

(24) $$x=\sum_{q=1}^{20}N_{q}\left(\xi ,\eta ,\zeta \right)x_{q},y=\sum_{q=1}^{20}N_{q}\left(\xi ,\eta ,\zeta \right)y_{q},z=\sum_{q=1}^{20}N_{q}\left(\xi ,\eta ,\zeta \right)z_{q}$$

The partial differentials of function u(x,y,z) with subject to axis ξ, η or ζ can be written as

(25) $$\begin{array}{l} \frac{\partial u}{\partial \xi }=\frac{\partial u}{\partial x}\frac{\partial x}{\partial \xi }+\frac{\partial u}{\partial y}\frac{\partial y}{\partial \xi }+\frac{\partial u}{\partial z}\frac{\partial z}{\partial \xi },\\ \frac{\partial u}{\partial \eta }=\frac{\partial u}{\partial x}\frac{\partial x}{\partial \eta }+\frac{\partial u}{\partial y}\frac{\partial y}{\partial \eta }+\frac{\partial u}{\partial z}\frac{\partial z}{\partial \eta },\\ \frac{\partial u}{\partial \zeta }=\frac{\partial u}{\partial x}\frac{\partial x}{\partial \zeta }+\frac{\partial u}{\partial y}\frac{\partial y}{\partial \zeta }+\frac{\partial u}{\partial z}\frac{\partial z}{\partial \zeta } \end{array}$$

Then the partial differentials of the function u(x,y,z) with respect to *x*, *y* and *z* are given by

(26) $$\begin{array}{l} \frac{\partial u}{\partial x}=\frac{1}{\left|J\right|}\left(\frac{\partial u}{\partial \xi }\beta_{11}+\frac{\partial u}{\partial \eta }\beta_{12}+\frac{\partial u}{\partial \zeta }\beta_{13}\right),\\ \frac{\partial u}{\partial y}=\frac{1}{\left|J\right|}\left(\frac{\partial u}{\partial \xi }\beta_{21}+\frac{\partial u}{\partial \eta }\beta_{22}+\frac{\partial u}{\partial \zeta }\beta_{23}\right),\\ \frac{\partial u}{\partial z}=\frac{1}{\left|J\right|}\left(\frac{\partial u}{\partial \xi }\beta_{31}+\frac{\partial u}{\partial \eta }\beta_{32}+\frac{\partial u}{\partial \zeta }\beta_{33}\right) \end{array}$$

in which $\beta_{ij}$ express the terms in the cofactor of Jacobi matrix *J*, and

(27) $$J=\left(\begin{array}{ccc} \frac{\partial x}{\partial \xi } & \frac{\partial x}{\partial \eta } & \frac{\partial x}{\partial \zeta }\\ \frac{\partial y}{\partial \xi } & \frac{\partial y}{\partial \eta } & \frac{\partial y}{\partial \zeta }\\ \frac{\partial z}{\partial \xi } & \frac{\partial z}{\partial \eta } & \frac{\partial z}{\partial \zeta } \end{array}\right)$$

Therefore, the first order partial differential in the physical domain can be written as

(28) $$u_{,x}=\left(\Delta_{11}D_{\xi }+\Delta_{12}D_{\eta }+\Delta_{13}D_{\zeta }\right)u=D_{x}u$$

(29) $$u_{,y}=\left(\Delta_{21}D_{\xi }+\Delta_{22}D_{\eta }+\Delta_{23}D_{\zeta }\right)u,=D_{y}u$$

(30) $$u_{,z}=\left(\Delta_{31}D_{\xi }+\Delta_{32}D_{\eta }+\Delta_{33}D_{\zeta }\right)u,=D_{z}u$$

in which

(31) Δij=(βij(1)|J(1)|0⋯00βij(2)|J(2)|⋯0⋯⋯⋯⋯00⋯βij(M)|J(M)|)

where $\beta_{ij}^{\left(1\right)}/\left|J^{\left(1\right)}\right|$ can be determined from Equation (27) at each node in the normalized domain, and the first order differentials matrix is determined by the Lagrange interpolation functions in normalized domain (|ξ|≤1,|η|≤1,|ζ|≤1).

### 3.4. Mapping Technology with 3D Blocks

**Figure 4:**
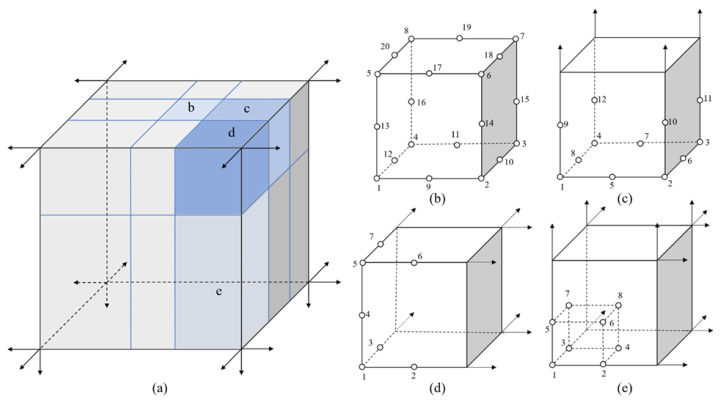
Mapping with four semi-infinite blocks: (**a**) semi-infinite model; (**b**) 20-seed-finite block; (**c**) 12-seed-one-infinite-edge block; (**d**) 7-seed-two-infinite-edge block; (**e**) 8-seed-three-infinite block.

For the semi-infinite structure shown in Figure 4a, the semi-infinite domain is divided into several subdomains with two 20-seed-finite blocks, two 12-seed-one-infinite-edge blocks, two 7-seed-two-infinite-edge blocks and two 8-seed-three-infinite-edge blocks as shown in figures from Figure 4b–e. The infinite blocks in different directions can be obtained by rotating the initial mapping function. The mapping function for the finite block and infinite blocks in a general form is written as

(32) $$N_{q}=Q(\xi ,\eta ,\zeta ,\xi_{q},\eta_{q},\zeta_{q})$$

where *q* is the seed number shown in Figure 4. The details of the mapping function and their partial differentials can be presented in Appendix A in different categories.

## 4. Formulations for Bimodular Material with Meshless FBM

The equilibrium equation, in the domain, gives

(33) ∇⋅σ+f=0

where ∇={∂/∂x,∂/∂y,∂/∂z}, and stress tensor

(34) $$\sigma =\left(\begin{array}{ccc} \sigma_{xx} & \sigma_{xy} & \sigma_{xz}\\ \sigma_{xy} & \sigma_{yy} & \sigma_{yz}\\ \sigma_{xz} & \sigma_{yz} & \sigma_{zz} \end{array}\right)\;\mathrm{and}\;f=\left(\begin{array}{c} f_{x}\\ f_{y}\\ f_{z} \end{array}\right)$$

in which $\sigma_{\alpha \beta },(\alpha ,\beta =x,y,z)$ denotes stress; $f_{\alpha }$ are body force. Substituting the constitutive equation, Equation (1), into the kinematic equation in Equation (33) without body forces yields

(35) $$\begin{array}{l} C_{11}u_{x}+C_{12}u_{y}+C_{13}u_{z}=0,\\ C_{21}u_{x}+C_{22}u_{y}+C_{23}u_{z}=0,\\ C_{31}u_{x}+C_{32}u_{y}+C_{33}u_{z}=0, \end{array}$$

where $u_{x},u_{y},u_{z}$ are vectors of nodal displacements, and $C_{ij},\left(i,j=1,\;2,\;3\right)$ are coefficients by the constitutive and equilibrium equations, and given by

(36) $$\begin{array}{l} C_{11}=Q_{11}D_{x}^{2}+2Q_{16}D_{x}D_{y}+2Q_{15}D_{x}D_{z}+Q_{66}D_{y}^{2}+2Q_{56}D_{y}D_{z}+Q_{55}D_{z}^{2},\\ C_{22}=Q_{66}D_{x}^{2}+2Q_{26}D_{x}D_{y}+2Q_{46}D_{x}D_{z}+Q_{22}D_{y}^{2}+2Q_{24}D_{y}D_{z}+Q_{44}D_{z}^{2},\\ C_{22}=Q_{55}D_{x}^{2}+2Q_{45}D_{x}D_{y}+2Q_{35}D_{x}D_{z}+Q_{44}D_{y}^{2}+2Q_{34}D_{y}D_{z}+Q_{33}D_{z}^{2},\\ C_{12}=C_{21}=Q_{16}D_{x}^{2}+(Q_{21}+Q_{66})D_{x}D_{y}+Q_{26}D_{y}^{2}+(Q_{25}+Q_{46})D_{y}D_{z}+Q_{45}D_{z}^{2},\\ C_{13}=C_{31}=Q_{15}D_{x}^{2}+(Q_{14}+Q_{56})D_{x}D_{y}+(Q_{13}+Q_{55})D_{x}D_{z}+Q_{46}D_{y}^{2}+(Q_{36}+Q_{45})D_{y}D_{z}+Q_{35}D_{z}^{2},\\ C_{23}=C_{32}=Q_{56}D_{x}^{2}+(Q_{25}+Q_{46})D_{x}D_{y}+(Q_{36}+Q_{45})D_{x}D_{z}+Q_{24}D_{y}^{2}+(Q_{23}+Q_{44})D_{y}D_{z}+Q_{34}D_{z}^{2}, \end{array}$$

where $Q_{ij},(i,j=1,\;2,\;\cdots ,6,\;Q_{ij}=Q_{ji})$ are the terms in elasticity matrix **Q** and given by

(37) $$Q=\left\{\begin{array}{cccccc} Q_{11} & Q_{12} & Q_{13} & Q_{14} & Q_{15} & Q_{16}\\ & Q_{22} & Q_{23} & Q_{24} & Q_{25} & Q_{26}\\ & & Q_{33} & Q_{34} & Q_{35} & Q_{36}\\ & & & Q_{44} & Q_{45} & Q_{46}\\ & \mathrm{Sym}. & & & Q_{55} & Q_{56}\\ & & & & & Q_{66} \end{array}\right\}.$$

Consider the following boundary conditions defined as

(38) $$\begin{array}{cc} t(x)=\bar{t}(x), & x\in \Gamma_{t}\\ u(x)=\bar{u}(x), & x\in \Gamma_{u} \end{array}$$

where $\bar{t}\left(x\right)$ and $\bar{u}\left(x\right)$ are given traction and displacement on the boundary, $\bar{t}\left(x\right)={\left\{{\bar{t}}_{x},{\bar{t}}_{y},{\bar{t}}_{z}\right\}}^{T}$, $\bar{u}\left(x\right)={\left\{{\bar{u}}_{x},{\bar{u}}_{y},{\bar{u}}_{z}\right\}}^{T}$. **x** is the collocation point on the boundary. Traction $\bar{t}(x)$ can be rewritten as

(39) $$\begin{array}{l} B_{11}u_{x}+B_{11}u_{x}+B_{11}u_{x}={\bar{t}}_{x},\\ B_{21}u_{x}+B_{22}u_{x}+B_{23}u_{x}={\bar{t}}_{y},\\ B_{31}u_{x}+B_{32}u_{x}+B_{33}u_{x}={\bar{t}}_{z} \end{array}$$

where matrix $B_{ij},\left(i,j=1,\;2,\;3\right)$ is associated with the boundary collocation point

(40) $$\begin{array}{l} B_{11}=D_{x}(Q_{11}n_{x}+Q_{16}n_{y}+Q_{15}n_{z})+D_{y}(Q_{16}n_{x}+Q_{66}n_{y}+Q_{56}n_{z})+D_{z}(Q_{15}n_{x}+Q_{56}n_{y}+Q_{55}n_{z}),\\ B_{22}=D_{x}(Q_{66}n_{x}+Q_{26}n_{y}+Q_{46}n_{z})+D_{y}(Q_{26}n_{x}+Q_{22}n_{y}+Q_{24}n_{z})+D_{z}(Q_{46}n_{x}+Q_{24}n_{y}+Q_{44}n_{z}),\\ B_{33}=D_{x}(Q_{55}n_{x}+Q_{45}n_{y}+Q_{35}n_{z})+D_{y}(Q_{45}n_{x}+Q_{44}n_{y}+Q_{34}n_{z})+D_{z}(Q_{35}n_{x}+Q_{34}n_{y}+Q_{33}n_{z}),\\ B_{12}=D_{x}(Q_{16}n_{x}+Q_{66}n_{y}+Q_{56}n_{z})+D_{y}(Q_{12}n_{x}+Q_{26}n_{y}+Q_{25}n_{z})+D_{z}(Q_{14}n_{x}+Q_{46}n_{y}+Q_{45}n_{z}),\\ B_{13}=D_{x}(Q_{15}n_{x}+Q_{56}n_{y}+Q_{55}n_{z})+D_{y}(Q_{14}n_{x}+Q_{46}n_{y}+Q_{45}n_{z})+D_{z}(Q_{13}n_{x}+Q_{36}n_{y}+Q_{35}n_{z}),\\ B_{21}=D_{x}(Q_{16}n_{x}+Q_{12}n_{y}+Q_{14}n_{z})+D_{y}(Q_{66}n_{x}+Q_{26}n_{y}+Q_{46}n_{z})+D_{z}(Q_{56}n_{x}+Q_{25}n_{y}+Q_{45}n_{z}),\\ B_{23}=D_{x}(Q_{56}n_{x}+Q_{25}n_{y}+Q_{45}n_{z})+D_{y}(Q_{46}n_{x}+Q_{24}n_{y}+Q_{44}n_{z})+D_{z}(Q_{36}n_{x}+Q_{23}n_{y}+Q_{34}n_{z}),\\ B_{31}=D_{x}(Q_{15}n_{x}+Q_{14}n_{y}+Q_{13}n_{z})+D_{y}(Q_{56}n_{x}+Q_{46}n_{y}+Q_{36}n_{z})+D_{z}(Q_{55}n_{x}+Q_{45}n_{y}+Q_{35}n_{z}),\\ B_{32}=D_{x}(Q_{56}n_{x}+Q_{46}n_{y}+Q_{36}n_{z})+D_{y}(Q_{25}n_{x}+Q_{24}n_{y}+Q_{23}n_{z})+D_{z}(Q_{35}n_{x}+Q_{34}n_{y}+Q_{33}n_{z}) \end{array}$$

where $n_{\alpha },(\alpha =x,y,z)$ is the boundary outwards normal. Therefore, 3 × *M* linear algebraic equations are obtained in total from Equations (33) and (38). In addition, on the interfaces between blocks, the following continued conditions should be taken into account

(41) $$\begin{array}{cc} u_{\alpha }^{\left(i\right)}-u_{\alpha }^{\left(j\right)}=0,t_{\alpha }^{\left(i\right)}+t_{\alpha }^{\left(j\right)}=0, & \left(\alpha =x,y,z\right) \end{array},$$

where $u_{\alpha }^{\left(i\right)}$ and $t_{\alpha }^{\left(i\right)}$ represent the displacement and traction on the interface between block *i* and block *j*. Finally, a set of linear algebraic equations is established the in global system as follows

(42) $$K_{\left[3M\times 3M\right]}U_{\left[3M\times 1\right]}=F_{\left[3M\times 1\right]},$$

Where **K** is the stiffness matrix, **U** is the vector of displacements and **F** is the vector consisting of the boundary value of the displacement, tractions and domain body forces. The following nonlinear iterative algorithm is adopted in this paper.

Step 1: m = 0, take either tensile or compressive modulus at all collocation points. Solve the global stiffness matrix to obtain the initial displacements, stresses and strains.

Step 2: Determine the principal stress $\sigma_{\alpha }$, $\sigma_{\beta }$, $\sigma_{\gamma }$ and the direction at each node. Then, determine the moduli, Poisson’s ratios ($E^{+},E^{-}$), ($v^{+},v^{-}$) and the constitutive matrix according from Equations (6)–(14).

Step 3: Modify the stiffness matrix **K** and vector **F** based on the current step. Solve the equations again to obtain the displacements, stresses and strains at each node.

Step 4: Calculate the average error from all collocation points

(43) $$\kappa =\frac{1}{M}\sum_{i=1}^{M}\left|U_{i}{}^{\left(m\right)}-U_{i}{}^{\left(m-1\right)}\right|$$

where $U_{i}{}^{\left(m\right)}$ presents the displacement at step *m*. if $\kappa <10^{-6}$, terminate the iteration and print out the result. Otherwise, let *m = m +* 1; go to step 2.

## 5. Numerical Examples

In this section, four examples are presented to demonstrate the accuracy of the meshless FBM with bimodular materials. A 3D tensile column with gravity is investigated in the first example. Then, FBM is applied to an arch bridge model, a single-layer semi-infinite model and a multi-layer pavement foundation under different loadings. All codes were written with Matlab (R2021b, The MathWorks, Inc., Natick, MA, USA).and Fortran in subroutine UMAT using ABAQUS (2019, Dassault Systèmes Simulia Corp., Vélizy-Villacoublay, France).

### 5.1. Tensile Column with Gravity

**Figure 5:**
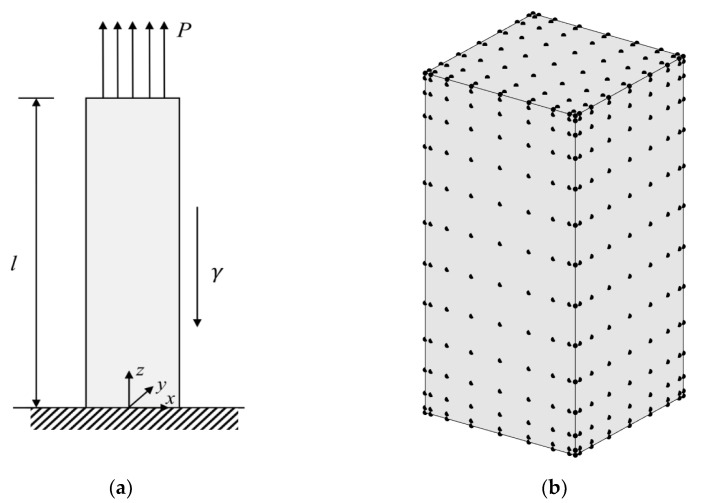
Model with a tensile load and gravity: (**a**) front view of model with load and constraint; (**b**) node distribution in physical domain for FBM.

Consider a gravitational column of the length *l* = 2; the dimension of the cross-section is normalized as 1 × 1, and the mass density γ = 2 as shown in Figure 5a. It is fixed on the bottom and a tensile force *P* of 2 units is applied to the top. It is assumed that a compression modulus is 5000 units, and the Poisson’s ratios in tension and compression is zero. The exact solution of the displacement [1] along the *z*-axis is given as

(44) $$\omega =\{\begin{array}{cc} \frac{Pz}{E^{-}}-\frac{\gamma }{E^{-}}\left(lz-\frac{1}{2}z^{2}\right), & z<c\\ \frac{\gamma }{2}\left[\frac{{\left(z-c\right)}^{2}}{E^{+}}-\frac{c^{2}}{E^{-}}\right], & z\geq c \end{array}$$

where c=l−P/γ. The numbers of node in *x*-axis and *y*-axis are 9, and in the *z*-axis is 14. The locations of node along different axes in the normalized domain are chosen

(45) $$\begin{array}{l} \xi_{i}=-cos\frac{\pi (i-1)}{N_{\xi }-1},i=1,2,\cdots ,N_{\xi };\\ \eta_{j}=-cos\frac{\pi (j-1)}{N_{\eta }-1},j=1,2,\cdots ,N_{\eta };\\ \zeta_{k}=-cos\frac{\pi (k-1)}{N_{\zeta }-1},k=1,2,\cdots ,N_{\zeta } \end{array}$$

**Figure 6:**
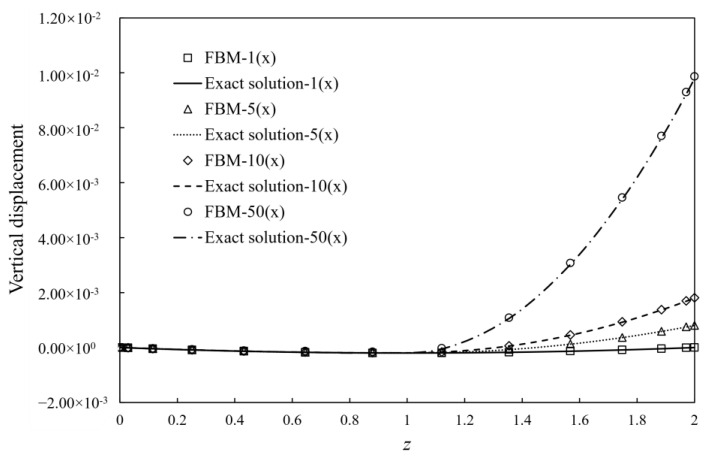
Vertical displacement variation along z-axis against with different ratios of tensile and compression moduli, where “n×”: $E^{-}/E^{+}=n$.

The total number of nodes for the FBM is 1134 (= 9 × 9 × 14), and 396 C3D20R elements are used in FEM. The node distribution of FBM is shown in Figure 5b. Comparison between the exact solution and FBM solution at point *z* = 1.96 and the number of iterations for convergence between FEM and FBM are presented in Table 1. With different ratios of tensile and compression modulus, the vertical displacement changes along the *z*-axis and exact solution are shown in Figure 6. Obviously, the FBM can give an accurate solution for the problem and shows a similar convergence rate when compared with the FEM method. To investigate the accuracy for different node density, the average relative errors are defined as

(46) $$\varepsilon =\frac{1}{M}\sum_{q=1}^{M}\left|\omega -\omega^{*}\right|$$

The numerical results presented in Table 2 demonstrate the average errors with iteration numbers of convergence over all collocation points when $E^{-}/E^{+}=10$. Observing the results in Table 2, it is evident that increasing the node density improves the degrees of accuracy, and convergency is easily approached in iterations when the node number $N_{\xi }$ is more than 3.

### 5.2. Arch Bridge in Bimodular Materials

**Figure 7:**
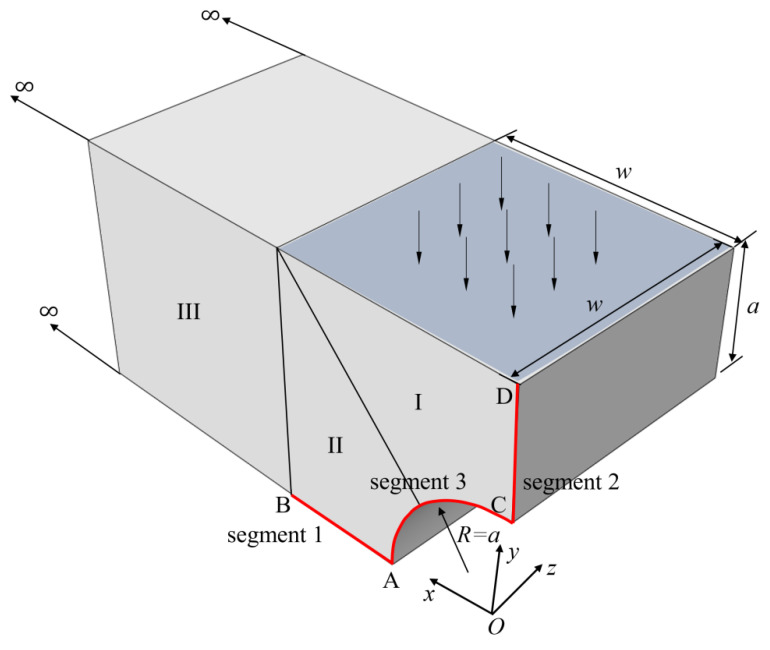
Half model of simplified arch bridge model for FBM.

Consider a simplified arch bridge as shown in Figure 7. Due to the symmetry of the structure, half of the model is taken for analysis. The radius of the arc is *a =* 1 unit. There is a vertical pressure load $p_{0}$ of 1 unit applied on the top, and the lengths in both *y*-axis and *x*-axis are *w*(=2*a*). The displacement $u_{y}$ is fixed on the bottom face (*y* = 0), and $u_{x}$ is zero on the surface *x =* 0. The ratios of Young’s moduli are selected as $E^{-}/E^{+}=1,\;2,\;5$, compression modulus $E^{-}=1$ unit and Poisson’s ratio $v^{-}=0.4$ in the computation procedure.

**Figure 8:**
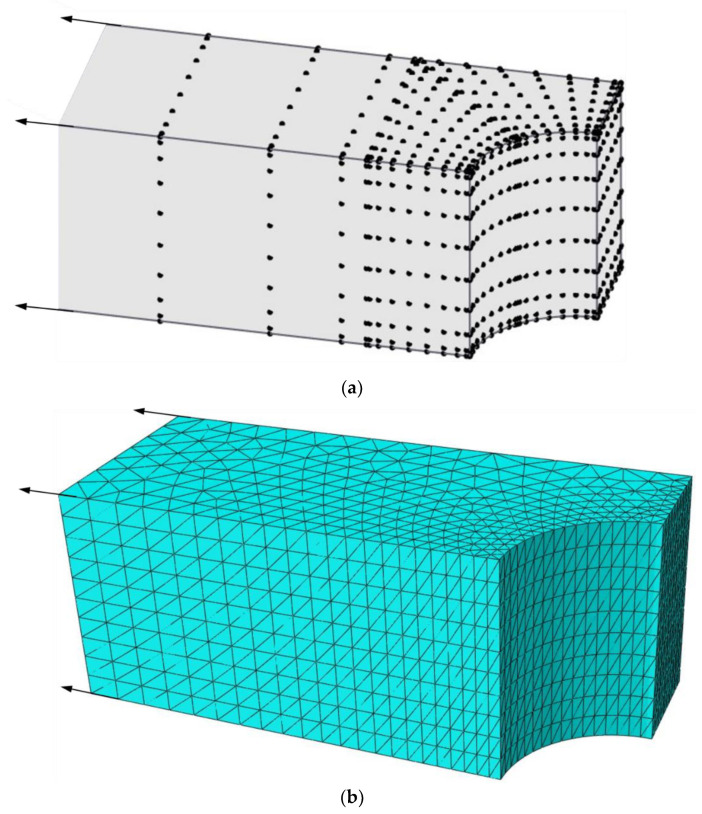
Half model for FBM and FEM: (**a**) nodes distribution for FBM; (**b**) finite element mesh for FEM.

**Figure 9:**
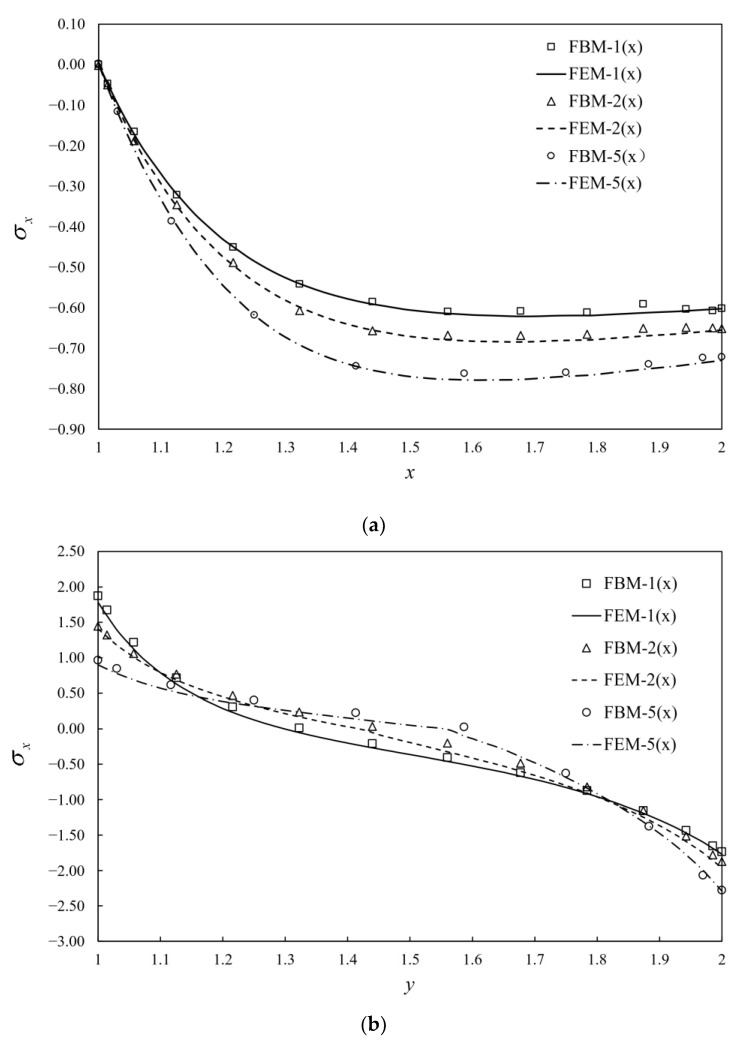

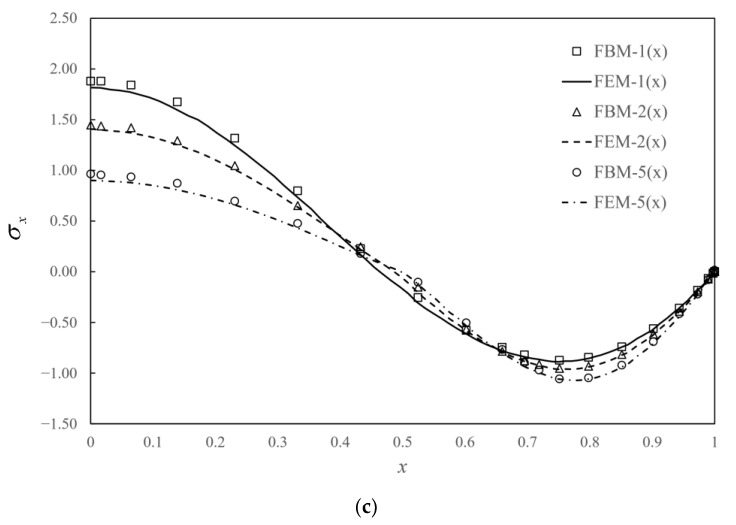
Dimensionless stress with 3 different ratios of Young’s moduli in tensile and compression along (**a**) AB; (**b**) CD; (**c**) AC and “n×”: $E^{-}/E^{+}=n$.

The bridge is divided into three blocks using FBM shown in Figure 7, where blocks I and II are finite blocks with 20-seed and block III is one semi-infinite block with a 12-seed-one-infinite-edge. In the discretization of each block, there are 12 and 14 collocation nodes along finite and infinite directions, respectively. The distribution of nodes along each axis is the same as Equation (45) in Section 5.1, as shown in Figure 8a. Stresses along two segments, AB and CD, shown in Figure 7 are plotted to illustrate the degree of accuracy. Simulation with FEM is complemented with 90,912 C3D10 elements as shown in Figure 8b. The length in the *x*-axis is *w* = 40 unit. The normalized stress $\sigma_{x}$ along AB, CD and AC by FBM and FEM is plotted in Figure 9 to show the difference between these two methods with bimodular materials. Reasonable agreements can clearly be observed. It is also noticed that there are several kinks in Figure 9 for stress distributions by the FEM due to the discontinuity of the Young’s modulus.

.

### 5.3. A Semi-Infinite Solid with Bimodular Materials

**Figure 10:**
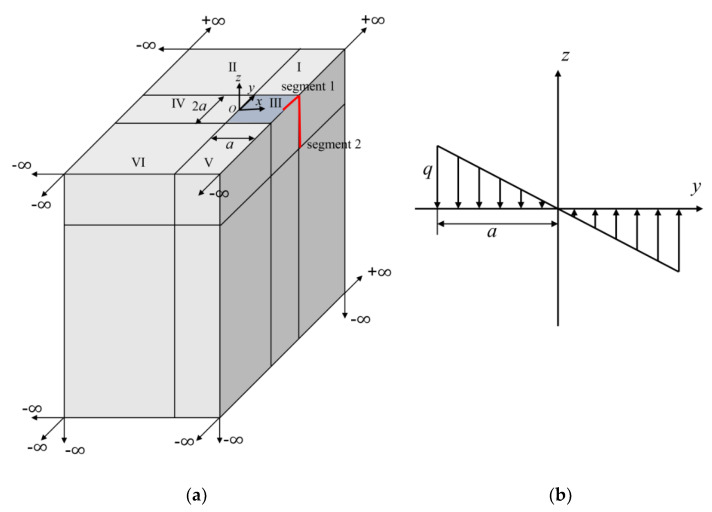
Semi-infinite model with linearly distributed vertical load: (**a**) semi-infinite model with 12 blocks by FBM; (**b**) side view from x-axis.

**Figure 11:**
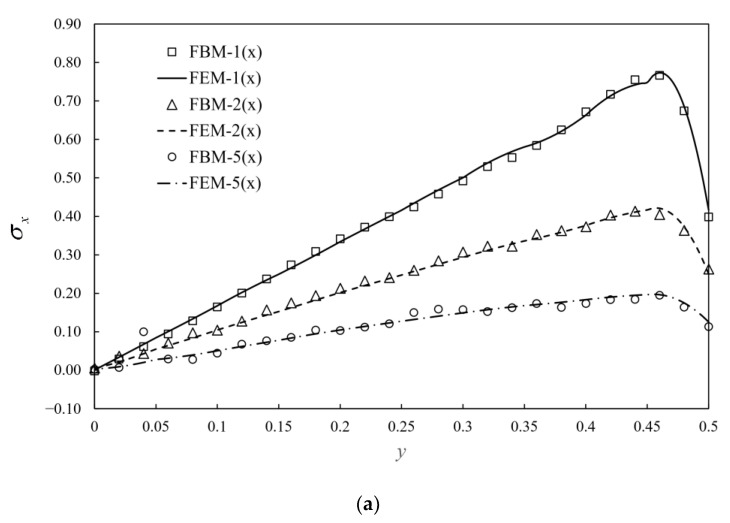

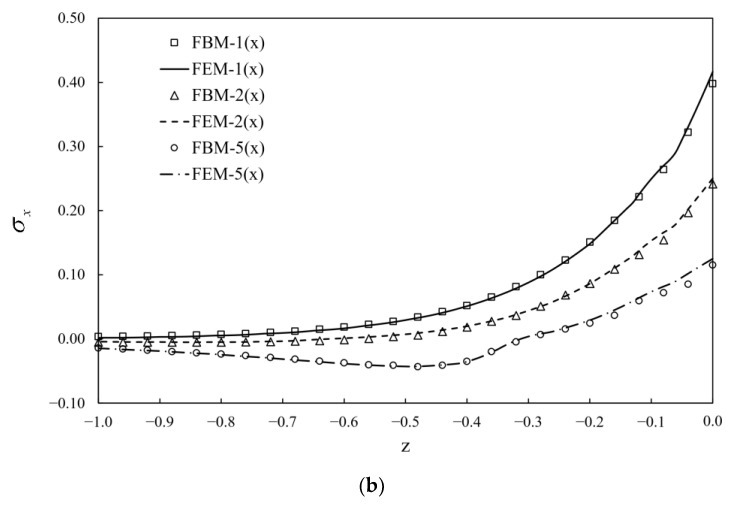
Normalized stress $\sigma_{x}$ given by FEM and FBM along: (**a**) AB; (**b**) AC and “n×”: $E^{-}/E^{+}=n$.

The semi-infinite structures are introduced to simulate soil foundations. Consider a semi-infinite body as shown in Figure 10a with the linear distributed vertical load in a square area of width 1 unit on the surface. The linear distributed load is plotted in Figure 10b with a unit maximum absolute value of *q* in compression and tension. Bimodular materials are selected with three different ratios of tensile and compressive moduli, as shown in Table 3. Due to the symmetry of the structure and loading, only a half model is analyzed as shown in Figure 10a. To accurately capture the stress near the loading area, the structure is subdivided into two layers. The first layer includes one 20-seed finite block III, three 12-seed-one-infinite-edge blocks I, IV, V and two 7-seed-two-infinite-edge block II and VI. However, in the second layer, one 12-seed-one-infinite-edge block, three 7-seed-two-infinite-edge blocks and two 8-seed-three-infinite-edges blocks are used. For each block, 9 collocation points are used on the finite edge and 12 points for the infinite edge. Normalized stress $\sigma_{x}$ along AB and AC are presented to demonstrate the accuracy of the FBM shown in Figure 11a,b versus the different ratios of tensile and compressive moduli, and Poisson ratios. In this example, FEM simulation is complemented by use of 362,484 C3D10 elements with dimensions of 20 units in length and height and 10 units in the width. A reasonable agreement was clearly achieved.

### 5.4. Multi-Layered Infinite Model with Bimodular Materials

**Figure 12:**
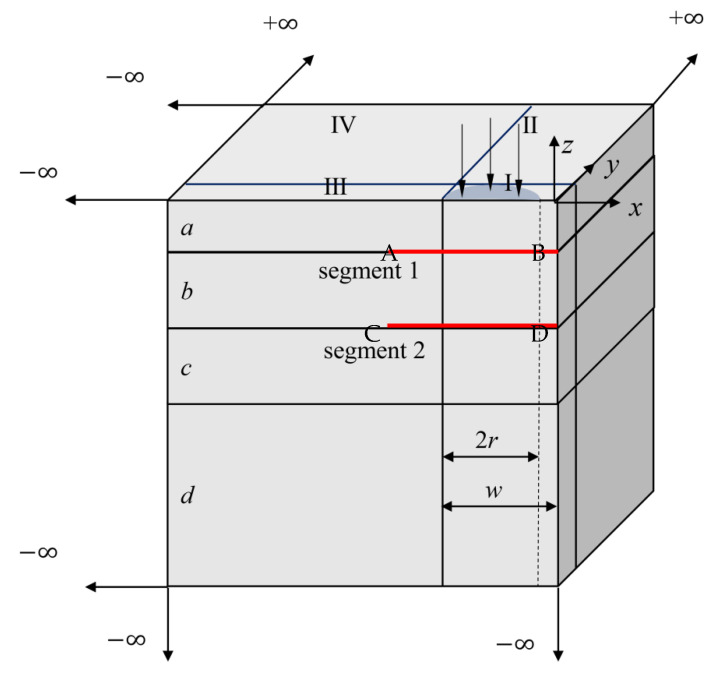
Quarter of meshless FBM with infinite block modeling.

Consider a multi-layered infinite structure, as shown in Figure 12, to simulate a highway pavement structure under two symmetric circular pressure loads. The pressure is assumed to be 0.7 MPa with a radius of 0.1065 m. The distance between two centers of loads is 0.3195 m. The model contains four layers; the first and second layers are bimodulus materials and the third and fourth layers are isotropic materials. The details of material parameters and dimensions of each layer are listed in Table 4. Again, due to the symmetry of the structure and load condition, quarter of the structure is analyzed as shown in Figure 12. During the numerical process, each layer is divided into four blocks. For the first layer, the top layer contains one 20-seed finite block, two 12-seed-infinite-edge blocks II and III and one 7-node-two-infinite-edge block IV. In the second and third layers, the same block distribution is applied as in the first layer. The bottom layer contains one 12-seed-one-infinite-edge block I, two 7-seed-two-infinite-edge blocks II and III and one 8-seed-three-infinite-edge block IV.

**Figure 13:**
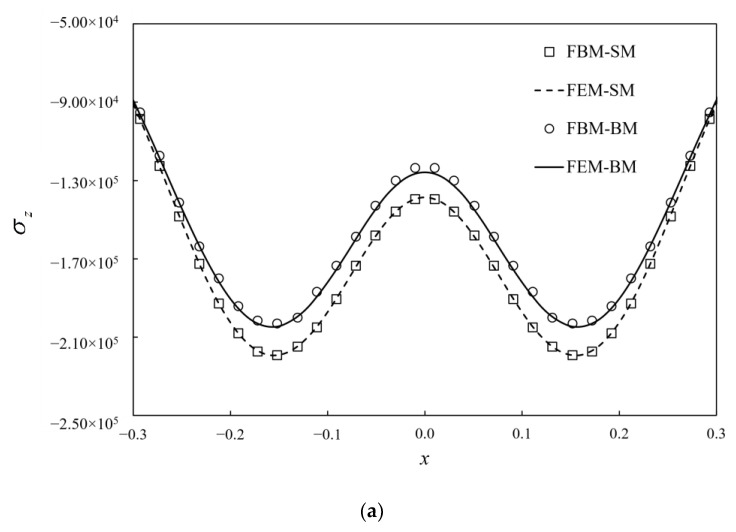

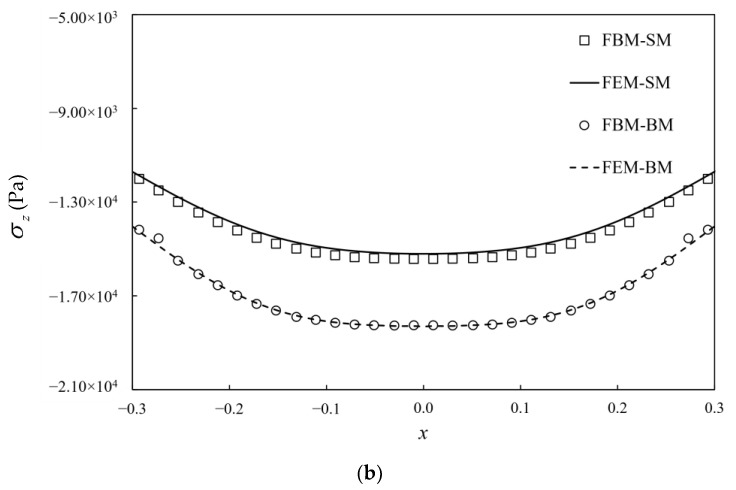
Stress $\sigma_{z}$ distribution and comparison with FEM on: (**a**) AB; (**b**) CD. SM indicates single Young’s modulus and BM indicates bimodular material.

**Figure 14:**
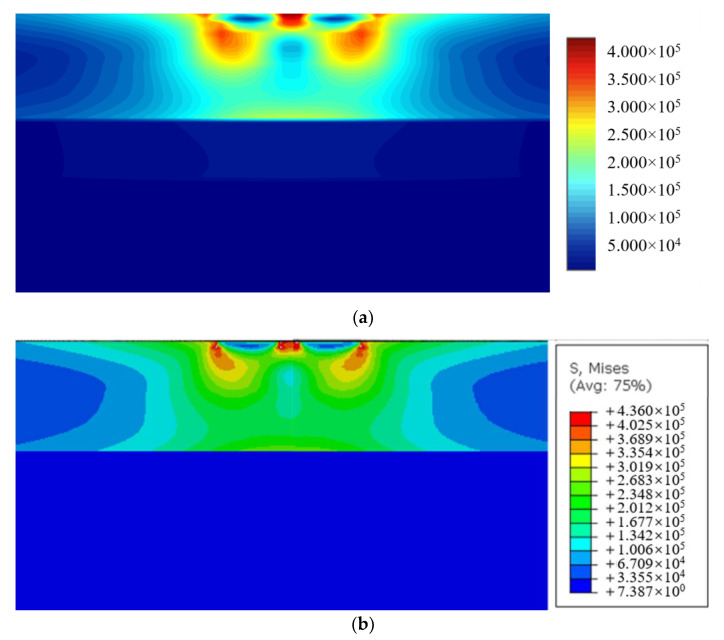
The contours of von Mises stress with bimodular materials for *y* = 0 by: (**a**) FBM; (**b**) FEM.

Like the node distributions in Section 5.3, the 8 seeds are used on the finite edges and 14 seeds on the infinite edges. The total number of collocation nodes by FBM is 12288. To validate the computational accuracy, the results of stresses $\sigma_{z}$ by FBM and FEM along segment AB and segment CD are compared in Figure 13. The contours of von Mises stress with bimodular materials on *y* = 0 are presented by using FBM in Figure 14. FEM is also used for analysis with no dimension of 20 × 20 × 20 and 127,760 C3D20R elements used in this example. It can be seen that the position of the maximum von Mises stress with these two methods is the almost the same, and the values are also very close to each other. In addition, the FBM results are smoother.

## 6. Conclusions

A meshless finite block method with infinite block analyzing three-dimensional solids of bimodular materials was demonstrated in this paper. A mapping technique was applied to determine the first order of derivatives. The 20-node finite block, 12-seed-one-edge-infinite block, 7-seed-two-edge-infinite block and 8-seed-three-edge-infinite block were introduced to simulate all semi-infinite domains. An iterative process for the meshless finite block method with a shear-modulus-complemented algorithm to solve bimodular problems was proposed. The numerical algorithm was validated with four examples. The finite element software ABAQUS was used for comparison. The following conclusions can be summarized: (1) FBM easily tackles nonlinear problems with semi-infinite boundaries; (2) a shear modulus algorithm efficiently and accurately describes the bimodular mechanical behavior of materials; (3) the proposed method shows efficiency and accuracy for semi-infinite problems with bimodular materials. Compared to FEM, FBM is more accurate with the same computational effort; (4) FBM can be applied to more complicated problems, such as 3D elastoplasticity, thermoelasticity and elastodynamics.

FE methods are rather general and efficient numerical tools to deal with complicated problems in engineering. However, as an alternative, the meshless finite block method with an infinite-mapping technique provides a new approach in solving unbounded bimodular material problems, with many advantages including efficiency and simplicity. As ABAQUS is a commercialized package, the CPU times used by different approaches are not comparable in this work. At present, dividing blocks is still a manual process in FBM; the versatility needs to be further improved with complex regional models. In future work, the FBM is expected to be extended to apply to more complicated problems, such as 3D elastoplasticity, thermoelasticity and elastodynamics.

## Figures and Tables

**Table 1:** Comparison of precision and convergence.

$E^{-}/E^{+}$	z = 1.96		Number of Iterations for Convergence	
	Exact Solution	FBM Solution	FEM	FBM
1	1.59 × 10^−5^	1.59 × 10^−5^	2	2
5	7.21 × 10^−4^	7.20 × 10^−4^	2	2
10	1.6 × 10^−3^	1.60 × 10^−3^	2	2
50	9.0 × 10^−3^	8.9 × 10^−3^	2	2

**Table 2:** Average errors ε for different node density with $E^{-}/E^{+}=10$.

Node Density $\left(N_{\xi }\times N_{\eta }\times N_{\zeta }\right)$	ε	Number of Iterationsfor Convergence
(3 × 3 × 6)	–	–
(4 × 4 × 8)	5.20 × 10^−5^	2
(5 × 5 × 10)	1.29 × 10^−5^	2
(7 × 7 × 14)	6.24 × 10^−6^	2
(9 × 9 × 18)	3.65 × 10^−6^	2
(11 × 11 × 22)	2.39 × 10^−6^	2

**Table 3:** Tensile and compressive modulus and Poisson’s ratio.

Case	Young’s Modulus $E^{+}/E^{-}$	Poisson’s Ratio $v^{+}/v^{-}$
1	1/1	0.4/0.4
2	0.5/1	0.2/0.4
3	0.2/1	0.08/0.4

**Table 4:** Dimensions, Young’s modulus and Poisson’s ratio for each layer.

Layer	Height (m)	$\mathrm{Young}’s\;\mathrm{Modulus}\;E^{+}/E^{-}\;(\mathrm{MPa})$	$\mathrm{Poisson}’s\;\mathrm{Ratio}\;v^{+}/v^{-}$
a	0.18	6000/9000	0.2/0.3
b	0.2	5000/8000	0.15625/0.25
c	0.2	300/300	0.35/0.35
d	∞	80/80	0.4

## Data Availability

The data presented in this study are available on request from the corresponding author.

## Appendix A

1. 20-node finite block

For this type of finite element, the physical domain is mapped to a cube with 20 seeds in coordination system (ξ,η,ζ) in the region |ξ|≤1, |η|≤1 and |ζ|≤1, as shown in Figure 4b. The mapping function can be written as follows [51]:

(A1) $$\begin{array}{cc} N_{i}=\frac{1}{8}(1+\xi_{i}\xi )(1+\eta_{i}\eta )(1+\zeta_{i}\zeta )(\xi_{i}\xi +\eta_{i}\eta +\zeta_{i}\zeta -2), & i=1,2,3,4,5,6,7,8 \end{array},$$

(A2) $$\begin{array}{cc} N_{i}=\frac{1}{4}(1-\xi^{2})(1+\eta_{i}\eta )(1+\zeta_{i}\zeta ), & i=9,11,17,19 \end{array},$$

(A3) $$\begin{array}{cc} N_{i}=\frac{1}{4}(1-\eta^{2})(1+\zeta_{i}\zeta )(1+\xi_{i}\xi ), & i=10,12,18,20 \end{array},$$

(A4) $$\begin{array}{cc} N_{i}=\frac{1}{4}(1-\zeta^{2})(1+\xi_{i}\xi )(1+\eta_{i}\eta ), & i=13,14,15,16 \end{array}.$$

Their partial differentials of the mapping function are listed below:

(A5) $$\begin{array}{l} \frac{\partial N_{i}}{\partial \xi }=\frac{\xi_{i}}{8}(1+\eta_{i}\eta )(1+\zeta_{i}\zeta )(2\xi_{i}\xi +\eta_{i}\eta +\zeta_{i}\zeta -1),\\ \frac{\partial N_{i}}{\partial \eta }=\frac{\eta_{i}}{8}(1+\xi_{i}\xi )(1+\zeta_{i}\zeta )(\xi_{i}\xi +2\eta_{i}\eta +\zeta_{i}\zeta -1),\\ \begin{array}{cc} \frac{\partial N_{i}}{\partial \zeta }=\frac{\zeta_{i}}{8}(1+\xi_{i}\xi )(1+\eta_{i}\eta )(\xi_{i}\xi +\eta_{i}\eta +2\zeta_{i}\zeta -1), & i=1,2,3,4,5,6,7,8 \end{array} \end{array}$$

(A6) $$\begin{array}{l} \frac{\partial N_{i}}{\partial \xi }=-\frac{1}{2}\xi (1+\eta_{i}\eta )(1+\zeta_{i}\zeta ),\\ \frac{\partial N_{i}}{\partial \eta }=\frac{1}{4}\eta_{i}(1-\xi^{2})(1+\zeta_{i}\zeta ),\\ \begin{array}{cc} \frac{\partial N_{i}}{\partial \xi }=\frac{1}{4}\zeta_{i}(1+\eta_{i}\eta )(1+\xi^{2}), & i=9,11,17,19 \end{array} \end{array}$$

(A7) $$\begin{array}{l} \frac{\partial N_{i}}{\partial \xi }=\frac{1}{4}\xi_{i}(1+\eta^{2})(1+\zeta_{i}\zeta ),\\ \frac{\partial N_{i}}{\partial \eta }=-\frac{1}{2}\eta (1+\xi_{i}\xi )(1+\zeta_{i}\zeta ),\\ \begin{array}{cc} \frac{\partial N_{i}}{\partial \xi }=\frac{1}{4}\zeta_{i}(1-\eta^{2})(1+\xi_{i}\xi ), & i=10,12,18,20 \end{array} \end{array}$$

(A8) $$\begin{array}{l} \frac{\partial N_{i}}{\partial \xi }=\frac{1}{4}\xi_{i}(1-\zeta^{2})(1+\eta_{i}\eta ),\\ \frac{\partial N_{i}}{\partial \eta }=\frac{1}{4}\eta_{i}(1-\zeta^{2})(1+\xi_{i}\xi ),\\ \begin{array}{cc} \frac{\partial N_{i}}{\partial \xi }=-\frac{1}{2}\zeta (1+\eta_{i}\eta )(1+\xi_{i}\xi ), & i=13,14,15,16 \end{array} \end{array}$$

2. 12-seed-one-edge-infinite block

In the normalized domain, the face of the upper side (ζ=1) is mapped to infinite area as shown in Figure 4c. The mapping functions [17] are

(A9) $$\begin{array}{cc} N_{i}=\frac{1}{2}(1+\xi_{i}\xi )(1+\eta_{i}\eta )(\xi_{i}\xi +\eta_{i}\eta -\zeta -2)/(1-\zeta ), & i=1,3,5,7 \end{array},$$

(A10) $$\begin{array}{cc} N_{i}=(1-\xi^{2})(1+\eta_{i}\eta )/(1-\zeta ), & i=2,6 \end{array},$$

(A11) $$\begin{array}{cc} N_{i}=(1+\xi_{i}\xi )(1-\eta^{2})/(1-\zeta ), & i=4,8 \end{array},$$

(A12) $$\begin{array}{cc} N_{i}=\frac{1}{4}(1+\xi_{i}\xi )(1+\eta_{i}\eta )(1+\zeta )/(1-\zeta ), & i=9,10,11,12 \end{array}.$$

The Cartesian coordinate system in the physical domain can be obtained

(A13) $$x=\sum_{k=1}^{12}N_{k}(\xi ,\eta ,\zeta )x_{k},y=\sum_{k=1}^{12}N_{k}(\xi ,\eta ,\zeta )y_{k},z=\sum_{k=1}^{12}N_{k}(\xi ,\eta ,\zeta )z_{k}.$$

The first-order partial differentials of Equations (A9)–(A12) are

(A14) $$\begin{array}{l} \frac{\partial N_{i}}{\partial \xi }=\frac{1}{2}\xi_{i}(1+\eta_{i}\eta )(-1+\eta_{i}\eta +2\xi_{i}\xi -\zeta )/(1-\zeta ),\\ \frac{\partial N_{i}}{\partial \eta }=\frac{1}{2}\eta_{i}(1+\xi_{i}\xi )(-1+\xi_{i}\xi +2\eta_{i}\eta -\zeta )/(1-\zeta ),\\ \begin{array}{cc} \frac{\partial N_{i}}{\partial \zeta }=\frac{1}{2}(1+\xi_{i}\xi )(1+\eta_{i}\eta )(-3+\eta_{i}\eta +\xi_{i}\xi -\zeta )/{\left(-1+\zeta \right)}^{2}, & i=1,3,5,7 \end{array} \end{array}$$

(A15) $$\begin{array}{l} \frac{\partial N_{i}}{\partial \xi }=2\xi (1+\eta_{i}\eta )/(1-\zeta ),\\ \frac{\partial N_{i}}{\partial \eta }=\eta_{i}(1-\xi^{2})/(1-\zeta ),\\ \begin{array}{cc} \frac{\partial N_{i}}{\partial \zeta }=(1+\eta_{i}\eta )(1-\xi^{2})/{\left(1-\zeta \right)}^{2}, & i=2,6 \end{array} \end{array}$$

(A16) $$\begin{array}{l} \frac{\partial N_{i}}{\partial \xi }=\xi_{i}(1+\eta^{2})/(1-\zeta ),\\ \frac{\partial N_{i}}{\partial \eta }=-2\eta (1+\xi_{i}\xi )/(1-\zeta ),\\ \begin{array}{cc} \frac{\partial N_{i}}{\partial \zeta }=(1-\eta^{2})(1+\xi_{i}\xi )/{\left(1-\zeta \right)}^{2}, & i=4,8 \end{array} \end{array}$$

(A17) $$\begin{array}{l} \frac{\partial N_{i}}{\partial \xi }=\frac{1}{4}\xi_{i}(1+\eta_{i}\eta )(1+\zeta )/(1-\zeta ),\\ \frac{\partial N_{i}}{\partial \eta }=\frac{1}{4}(1+\xi_{i}\xi )\eta_{i}(1+\zeta )/(1-\zeta ),\\ \begin{array}{cc} \frac{\partial N_{i}}{\partial \zeta }=\frac{1}{2}(1+\eta_{i}\eta )(1+\xi_{i}\xi )/{\left(-1+\zeta \right)}^{2}, & i=9,10,11,12 \end{array} \end{array}$$

3. 7-seed-two-edge-infinite block

In this case, two edges (ξ=1,η=1) in the normalized domain is mapped to infinite place as shown in Figure 4d. The shape functions [17,52] are

(A18) $$\begin{array}{l} N_{1}=(1-\xi )(-5-\xi -\eta -4\zeta +3\xi \eta )/(2\alpha ),\\ N_{2}=\frac{\left(1-\zeta )(1+\xi \right)}{\left[2(1-\xi )\right]},\\ N_{3}=\frac{\left(1-\zeta )(1+\eta \right)}{\left[2(1-\xi )\right]},\\ N_{4}=\frac{4(1-\zeta^{2})}{\alpha },\\ N_{5}=(1+\zeta )(-5-\xi -\eta +4\zeta +3\xi \eta )/(2\alpha ),\\ N_{6}=\frac{\left(1+\zeta )(1+\xi \right)}{\left[2(1-\xi )\right]},\\ N_{7}=\frac{\left(1-\zeta )(1+\xi \right)}{\left[2(1-\eta )\right]}, \end{array}$$

in which α=(1−ξ)(1−η) with coordinate transformation

(A19) $$x=\sum_{k=1}^{7}N_{k}(\xi ,\eta ,\zeta )x_{k},y=\sum_{k=1}^{7}N_{k}(\xi ,\eta ,\zeta )y_{k},z=\sum_{k=1}^{7}N_{k}(\xi ,\eta ,\zeta )z_{k}.$$

Their partial differential with respect to ξ, η and ζ are given as follows:

(A20) $$\begin{array}{l} \frac{\partial N_{1}}{\partial \xi }=(1-\zeta )(3-\eta +2\zeta )/\beta ,\\ \frac{\partial N_{2}}{\partial \xi }=(1-\zeta )/{\left(1-\xi \right)}^{2},\\ \frac{\partial N_{3}}{\partial \xi }=\frac{\partial N_{7}}{\partial \xi }=0,\\ \frac{\partial N_{4}}{\partial \xi }=4(1-\zeta^{2})/\beta ,\\ \frac{\partial N_{5}}{\partial \xi }=(1+\xi )(-3+\eta +2\zeta )/\beta ,\\ \frac{\partial N_{6}}{\partial \xi }=(1+\zeta )/{\left(1-\xi \right)}^{2}, \end{array}$$

(A21) $$\begin{array}{l} \frac{\partial N_{1}}{\partial \eta }=(1-\zeta )(-3+\xi -2\zeta )/\gamma ,\\ \frac{\partial N_{2}}{\partial \eta }=\frac{\partial N_{6}}{\partial \eta }=0,\\ \frac{\partial N_{3}}{\partial \eta }=(1-\xi )/{\left(1-\eta \right)}^{2},\\ \frac{\partial N_{4}}{\partial \eta }=4(1-\zeta^{2})/\gamma ,\\ \frac{\partial N_{5}}{\partial \eta }=(1+\zeta )(-3+\eta +2\zeta )/\gamma ,\\ \frac{\partial N_{7}}{\partial \eta }=(1+\zeta )/{\left(1-\eta \right)}^{2}, \end{array}$$

(A22) $$\begin{array}{l} \frac{\partial N_{1}}{\partial \zeta }=(1-\xi +\eta -3\xi \eta +8\zeta )/(2\alpha ),\\ \frac{\partial N_{2}}{\partial \zeta }=-(1+\xi )/[2(1-\xi )],\\ \frac{\partial N_{3}}{\partial \zeta }=-(1+\eta )/[2(1-\eta )],\\ \frac{\partial N_{4}}{\partial \zeta }=-8\zeta /\alpha ,\\ \frac{\partial N_{5}}{\partial \zeta }=(-1-\xi -\eta +3\xi \eta +8\zeta )/(2\alpha ),\\ \frac{\partial N_{6}}{\partial \zeta }=(1+\xi )/[2(1-\xi )]\\ \frac{\partial N_{7}}{\partial \zeta }=(1+\eta )/[2(1-\eta )], \end{array}$$

where α=(1−ξ)(1−η), β=α(1−ξ), γ=α(1−η).

4. 8-seed-three-edge-infinite block

This type of infinite element is extended from Lagrangian 27-node brick, which is shown in Figure 4e. Three directions (ξ=1,η=1,ζ=1) in the normalized domain are mapped to infinity. The shape functions [17,52] are simplified

(A23) $$\begin{array}{l} N_{1}=-8\xi \eta \zeta /\alpha ,\\ N_{2}=4\eta \zeta (1+\xi )/\alpha ,\\ N_{3}=4\xi \zeta (1+\eta )/\alpha ,\\ N_{4}=-2\zeta (1+\xi )(1+\eta )/\alpha ,\\ N_{5}=4\xi \eta (1+\zeta )/\alpha ,\\ N_{6}=-2\eta (1+\xi )(1+\zeta )/\alpha ,\\ N_{7}=-2\xi (1+\eta )(1+\zeta )/\alpha ,\\ N_{8}=(1+\xi )(1+\eta )(1+\zeta )/\alpha , \end{array}$$

where α=(1−ξ)(1−η)(1−ζ) with coordinate transformation

(A24) $$x=\sum_{k=1}^{8}N_{k}(\xi ,\eta ,\zeta )x_{k},y=\sum_{k=1}^{8}N_{k}(\xi ,\eta ,\zeta )y_{k},z=\sum_{k=1}^{8}N_{k}(\xi ,\eta ,\zeta )z_{k}.$$

Their partial differential with respect to ξ, η and ζ are listed as follows:

(A25) $$\begin{array}{l} \frac{\partial N_{1}}{\partial \xi }=-8\eta \zeta /\beta ,\\ \frac{\partial N_{2}}{\partial \xi }=8\eta \zeta /\beta ,\\ \frac{\partial N_{3}}{\partial \xi }=4\zeta (1+\eta )/\beta ,\\ \frac{\partial N_{4}}{\partial \xi }=-4\zeta (1+\eta )/\beta ,\\ \frac{\partial N_{5}}{\partial \xi }=-4\eta (1+\zeta )/\beta ,\\ \frac{\partial N_{6}}{\partial \xi }=4\eta (1+\zeta )/\beta ,\\ \frac{\partial N_{7}}{\partial \xi }=-2(1+\eta )(1+\zeta )/\beta ,\\ \frac{\partial N_{8}}{\partial \xi }=2(1+\eta )(1+\zeta )/\beta , \end{array}$$

(A26) $$\begin{array}{l} \frac{\partial N_{1}}{\partial \eta }=-8\xi \zeta /\gamma ,\\ \frac{\partial N_{2}}{\partial \eta }=4\zeta (1+\xi )/\gamma ,\\ \frac{\partial N_{3}}{\partial \eta }=8\xi \zeta /\gamma ,\\ \frac{\partial N_{4}}{\partial \eta }=-4\zeta (1+\xi )/\gamma ,\\ \frac{\partial N_{5}}{\partial \eta }=4\xi (1+\zeta )/\gamma ,\\ \frac{\partial N_{6}}{\partial \eta }=-2(1+\xi )(1+\zeta )/\gamma ,\\ \frac{\partial N_{7}}{\partial \eta }=-4\xi (1+\zeta )/\gamma ,\\ \frac{\partial N_{8}}{\partial \eta }=2(1+\xi )(1+\zeta )/\gamma , \end{array}$$

(A27) $$\begin{array}{l} \frac{\partial N_{1}}{\partial \zeta }=-8\xi \eta /\delta ,\\ \frac{\partial N_{2}}{\partial \zeta }=4\eta (1+\xi )/\delta ,\\ \frac{\partial N_{3}}{\partial \zeta }=4\xi (1+\eta )/\delta ,\\ \frac{\partial N_{4}}{\partial \zeta }=-2(1+\xi )(1+\eta )/\delta ,\\ \frac{\partial N_{5}}{\partial \zeta }=8\xi \eta /\delta ,\\ \frac{\partial N_{6}}{\partial \zeta }=-4\eta (1+\xi )/\delta ,\\ \frac{\partial N_{7}}{\partial \zeta }=-4\xi (1+\eta )/\delta ,\\ \frac{\partial N_{8}}{\partial \zeta }=2(1+\xi )(1+\eta )/\delta , \end{array}$$

in which β=α(1−ξ), γ=α(1−η) and δ=α(1−ζ).
